# Evaluation of Current Equine Influenza Vaccination Protocols Prior to Shipment, Guided by OIE Standards

**DOI:** 10.3390/vaccines8010107

**Published:** 2020-02-29

**Authors:** Ann Cullinane, Jacinta Gahan, Cathal Walsh, Manabu Nemoto, Johanna Entenfellner, Cecilia Olguin-Perglione, Marie Garvey, Tao Qi Huang Fu, Monica Venner, Takashi Yamanaka, María Barrandeguy, Charlene Judith Fernandez

**Affiliations:** 1 Virology Unit, The Irish Equine Centre, Naas, Co. Kildare, W91 RH93 Johnstown, Ireland; JGahan@irishequinecentre.ie (J.G.); nemoto_manabu@equinst.go.jp (M.N.); MGarvey@irishequinecentre.ie (M.G.); 2 Department of Mathematics and Statistics, University of Limerick, V94 T9PX Limerick, Ireland; Cathal.Walsh@ul.ie; 3 Equine Research Institute, the Japan Racing Association, 1400-4 Shiba, Shimotsuke, Tochigi 329-0412, Japan; yamanaka@equinst.go.jp; 4 Equine Clinic, School of Veterinary Medicine, Bischofsholer Damm 15, 30173 Hannover, Germany; johannaentenfellner@icloud.com; 5 Instituto Nacional de Tecnología Agropecuaria (INTA), Instituto de Virología, De Los Reseros y Dr. Nicolás Repetto S/N, Hurlingham, Buenos Aires B1686IGC, Argentina; olguin.cecilia@inta.gob.ar; 6 Centre for Animal and Veterinary Sciences, Professional and Scientific Services, Animal and Veterinary Service, National Parks Board, 1 Cluny Road, Singapore 259569, Singapore; Huang_Fu_Tao_Qi@nparks.gov.sg (T.Q.H.F.); Charlene_FERNANDEZ@nparks.gov.sg (C.J.F.); 7 Pferdeklinik Destedt GmbH, Destedt, Trift 4, 38162 Cremlingen, Germany; MVenner@gmx.de; 8 Escuela de Veterinaria, Universidad del Salvador, Champagnat 1599, Ruta Panamericana km 54.5 Pilar, Buenos Aires B1630AHU, Argentina; barrandeguy.maria@inta.gob.ar

**Keywords:** equine influenza, vaccination, shipment, OIE, FEI, IFHA, harmonization, age, horses

## Abstract

To facilitate the temporary importation of horses for competition and racing purposes, with a minimum risk of transmitting equine influenza, the World Organisation for Animal Health (Office International des Epizooties, or OIE), formally engaged in a public–private partnership with the Federation Equestre Internationale (FEI) and the International Federation for Horseracing Authorities (IFHA) to establish, within the context of existing OIE standards, a science-based rationale to identify the ideal time period for equine influenza vaccination prior to shipment. Field trials using vaccines based on different technologies were carried out on three continents. The antibody response post-booster vaccination at intervals aligned with the different rules/recommendations of the OIE, FEI, and IFHA, was monitored by single radial haemolysis. It was determined that 14 days was the optimum period necessary to allow horses adequate time to respond to booster vaccination and for horses that have previously received four or more doses of vaccine and are older than four years, it is adequate to allow vaccination within 180 days of shipment. In contrast, the results indicate that there is a potential benefit to younger (four years old or younger) horses in requiring booster vaccination within 90 days of shipment, consistent with the current OIE standard.

## 1. Introduction

Equine influenza (H3N8) viruses are highly contagious, particularly in immunologically naïve populations [1]. The first incursion in Australia resulted in the infection of approximately 75,000 horses on 9,400 premises in New South Wales and Queensland, and at the very early stage of the epidemic up to 98 premises became infected through the movement of horses from two equestrian competition events [2,3]. The economic losses to the equine industry, in addition to the implementation of an immediate national standstill of horses followed by risk-based movement controls, laboratory testing, strategic vaccination of over 136,000 horses, and other measures that contributed to an effective eradication programme, are estimated to have cost billions of dollars [4]. Similar equine influenza virus incursions in Hong Kong [5], Japan [6], South Africa [7,8], Turkey [9], Malaysia [10] and other countries have also resulted in economic loss, due to movement restrictions and cancellation of equestrian events. More recently, in 2018 and 2019, widespread outbreaks in South America [11] and Africa affected thousands of horses and donkeys, respectively [12]. In 2019, many European countries experienced an increase in equine influenza activity [13], and in the United Kingdom horse racing was cancelled for six days, with media reports of revenue loss in excess of £100 million. 

In a systematic review by Dominguez et al. [14], equine influenza was identified as the equine pathogen most frequently responsible for disease events post-importation. The international spread of equine influenza is frequently associated with the movement of horses from endemic areas, such as Europe and North America [5,7,8,9,15]. The Australian outbreak appears to have been introduced by thoroughbred horses from Japan, the virus having recently arrived there from the United States [16]. The majority of importing countries require that horses are vaccinated against equine influenza, and sporting bodies implement mandatory vaccination policies to minimise disruption to equestrian events. However, the vaccines do not afford sterile immunity, and virus incursions are frequently associated with the importation of subclinically infected vaccinated horses [5,6,7,8,16,17]. In order to decrease this risk, it is essential that the vaccination policies implemented are science-based and targeted towards minimising virus shedding.

To facilitate the temporary importation of horses for competition and racing purposes, with a minimum risk of transmitting infectious diseases like equine influenza, the World Organisation for Animal Health (Office International des Epizooties, or OIE) formally engaged in a public–private partnership with the Federation Equestre Internationale (FEI) and the International Federation for Horseracing Authorities (IFHA). The main aim of this initiative was to establish, within the context of the existing OIE standards, new standards and guidelines relating to infectious diseases, such as equine influenza. According to the FEI veterinary rules and the IFHA guidelines, competing horses must be vaccinated against equine influenza, but their recommendations are not harmonized and not in line with OIE standards, as documented in the Terrestrial Animal Health Code [18]. As industry partners, the FEI and IFHA provided support to the OIE to conduct this project, the aim of which was to provide a science-based rationale for identifying the ideal time period for equine influenza vaccination prior to shipment. Two international field trials were carried out, using vaccines based on different technologies. The aim of the first trial was to determine the mandatory interval between booster vaccination and shipment necessary to allow the horse adequate time to respond to booster vaccination. The aim of the second trial was to determine the durability of the antibody response induced by booster vaccination, and therefore the appropriate maximum interval between booster vaccination and shipment.

## 2. Materials and Methods

### 2.1. Study Design

Table 1 summarises the FEI, IFHA, and OIE recommendations for vaccination prior to export or competition. In an effort to harmonise these recommendations, two international field trials were conducted. The focus of Trial A was to determine the time necessary for the horses to respond to booster vaccination, i.e., how close to shipment they could be vaccinated. Thus, horses received their booster vaccination in accordance with their usual vaccination schedule, and their response was monitored at 7 days (FEI), 14 days (IFHA), and 21 days (OIE) post-booster. The focus of Trial B was to determine how long before shipment horses could be vaccinated and maintain a high level of antibodies at the time of shipment. Thus, horses received their booster vaccination in accordance with their usual vaccination schedule, and their response was monitored at 60 days (IFHA), 90 days (OIE), and 180 days (FEI) post-booster. In both trials, the response to vaccination was monitored using the single radial haemolysis (SRH) test, as the correlation between SRH antibodies and protection against infection (virological protection) and disease (clinical protection) is well established [19,20,21].

### 2.2. Horses

The selection of horses was at the discretion of the participating countries that volunteered to assist with either or both trials in the target population, i.e., racehorses or sport horses. Experimental animals were not eligible for inclusion. In all countries, the horses recruited to the study were due their booster vaccination as part of routine preventative health measures, and in accordance with the regulations of the relevant authorities. The attending veterinary surgeon administered the customary vaccine, collected the blood samples, and monitored the health status of the horses in their care throughout the study period. The inclusion of the horses in the trials was dependent on the good will and cooperation of the horse owners/managers, the veterinary surgeons and the support staff. Germany performed studies for both Trials A and B in the same group of horses, Argentina and Japan performed three studies (two for Trial A and one for Trial B in Argentina; two for Trial B and one for Trial A in Japan) in different groups of horses, and Singapore opted to perform one study for Trial B. In each country, participation in the study (AD/SR/2015/1885 2.3.) was approved by the appropriate institutional review board.

Of the 194 horses recruited for Trial A, 103 were in Germany, 64 were in Argentina and 27 were in Japan. In Germany, the horses were young sport horses ranging in age from two to seven years old (median: three years). They received a dose of vaccine approximately six months prior to the study. They received a total of 5 to 16 (mean = 7.5 ± 2.2) doses of vaccine. In Japan, the horses were sport horses ranging in age from 6 to 20 (median: 13 years), and they had been vaccinated six months prior to the study. While under the care of the Japanese Racing Association (JRA), the horses received at least 4 to 40 doses of vaccine (mean = 18.7 ± 9.5) in approximately six monthly intervals. No vaccination history was available prior to JRA ownership. In Argentina, 30 sport horses ranged in age from 4 to 17 (median: eight years), and 30 of 34 racehorses ranged in age from three to five (median: three years). The age of the remaining four racehorses was not available. Individual vaccination histories were not available for the Argentine horses, but it is mandatory in Argentina that both racehorses and sport horses are vaccinated four times a year. The horses recruited to the study had received a dose of vaccine three months prior to the study.

Of the 226 horses recruited for Trial B, 79 were in Germany, 33 were in Argentina, 74 were in Japan and 40 were in Singapore. In Germany, the horses were young sport horses ranging in age from two to seven years old (median: four years). They had received a dose of vaccine approximately six months prior to the study. They received a total of 5 to 16 (mean = 7.9 ± 2.2) doses of vaccine. In Japan, 43 three-year-old racehorses and 31 sport horses ranging in age from 6 to 20 (median: 13 years) were recruited to the study. Both groups had received a dose of vaccine approximately six months prior to the study. The racehorses received a total of six doses of vaccine, and the sport horses received at least 4 to 40 doses of vaccine (mean = 19.2 ± 9.2). There were no vaccination records available prior to JRA ownership; thus, the numbers cited may be an underestimation of the vaccines received. In Argentina, 33 sport horses ranged in age from 4 to 21 (median: 11 years). They received a total of 4 to 26 (mean = 13.3 ± 6.5) doses of vaccine, and had received their last vaccine three months prior to the study. In Singapore, 40 racehorses ranged in age from 4 to 12 (median: five years). Vaccination records for 28 of these horses indicated that they received a total of 2 to 19 (mean = 5.8 ± 3.8) doses of vaccine, and had received their last vaccine six months prior to the study.

### 2.3. Vaccines

For Trial A in Germany, the horses were randomly allocated one of three vaccines, 34 were vaccinated with a subunit vaccine—namely, Equip FT (Zoetis, Parsippany-Troy Hills, NJ, United States), 34 with the canary pox recombinant vaccine ProteqFlu TE (Merial S.A.S./Boehringer Ingelheim,29 Avenue Tony Garnier, 69007, Lyon, France), and 35 with the whole inactivated virus vaccine Duvaxyn IE T Plus (Elanco Animal Health, Eli Lilly Ltd., Priestly Road, Basingstoke, RG24 9NL, United Kingdom). In Japan, the 27 horses were vaccinated with a nationally produced, whole inactivated vaccine (Nisseiken, Shin-machi, Ome, Tokyo 198-0024, Japan). In Argentina, 34 racehorses were vaccinated with the inactivated vaccine Fluvac Innovator (Zoetis), and 30 sport horses were vaccinated with the nationally produced, inactivated vaccine INFLUQIM (Biochemiq, Del Cañon 2690, Moreno, Buenos Aires, Argentina).

For Trial B, the horses in Germany had been included in Trial A, and thus 26 had been vaccinated with a subunit vaccine—namely Equip FT (Zoetis), 23 with the canary pox recombinant vaccine ProteqFlu TE (Boehringer Ingelheim/Merial), and 30 with the whole inactivated virus vaccine Duvaxyn IE T Plus (Elanco). In Japan, all horses were vaccinated with a whole inactivated Japanese vaccine (Nisseiken). In Argentina, all horses were vaccinated with the inactivated vaccine Fluvac Innovator (Zoetis), and in Singapore the horses were vaccinated with the recombinant vaccine ProteqFlu TE (Boehringer Ingelheim/Merial).

### 2.4. Sample Collection

For Trial A, clotted blood samples were collected on the day of vaccination, as well as 7, 14, and 21 days post-vaccination. These sampling days were aligned with the rules and recommendations of the FEI (7 days), IFHA (14 days), and OIE (21 days).

For Trial B, clotted blood samples were collected on the day of vaccination and day 21 (Germany), day 30 (Japan, Argentina, Singapore), days 60 and 90 (Germany, Japan, Argentina, Singapore), and day 180 post-vaccination (Germany, Japan, Argentina). The sampling days of 60, 90, and 180 were aligned with the rules and recommendations of the IFHA (60 days), FEI (6 months +21 days grace period), and OIE (90 days).

As these were field trials conducted in the target population, some of the horses could not be sampled at one or more of the sampling times for operational reasons, or they were lost to the study because they were sold or removed from the premises. In Germany, the same horses were used for Trial A and Trial B. In order to reduce labor and animal handling, it was agreed that the sample collected at 21 days for Trial A could also serve to monitor the response to vaccination for Trial B, the aim of which was to compare the antibody levels at three time points, i.e., days 60, 90, and 180 days post-vaccination.

### 2.5. Serology

Antibodies against two H3N8 viruses--A/equine/Meath/2007, a representative of Clade 2 of the Florida sublineage, and A/equine/South Africa/4/2003, a representative of Clade 1 of the Florida sublineage—were measured using the SRH test, as previously described [22]. The area of haemolysis resulting from the lysis of equine influenza antigen-coated sheep red blood cells by the antibody in the test sera were expressed in mm^2^. Mean H3N8 antibody values were calculated from SRH results obtained, and seroconversion was defined as an increase in antibody level of ≥25 mm^2^ [23]. Similarly, a significant decline in antibody titre was defined as a decrease of ≥25 mm^2^. The laboratory investigator was blind to vaccine allocation to individual horses.

### 2.6. Statistical Analysis

All statistical analysis was carried out on the base distribution of the open-source package R version 3.1.1 [24], with no additional specific packages used. The R code used for analyses is available from the authors on request. A significance level of *p* < 0.05 was used for all statistical tests. Throughout the report, where there were missing values, the analysis was carried out using case-wise deletion. A case-wise deletion strategy was used in order to ensure the values at each timepoint were based on the same cohort of animals; the reason for missing observations is discussed in the section on sample collection above. Plots and analyses contain >150 horses unless otherwise specified. When comparing changes in titre between timepoints, pairwise *t*-tests were used. In examining the association of multiple covariates with the response to vaccination and subsequent waning, a multivariate linear model was fitted, with backwards stepwise elimination where appropriate. In these multivariate analyses, models were fitted to summarise the impact of vaccination on the change in titre from the baseline in different animals. The variables entered into the model included age, vaccine type, and number of previous vaccinations. Separate models were fitted, including age and number of previous vaccinations, because of confounding between these variables, as discussed below.

## 3. Results

In accordance with previously established correlates of the protection SRH titres >150 mm^2^ were interpreted as consistent with virological protection, and titres >85 mm^2^ but <150 mm^2^ were interpreted as consistent with clinical protection against antigenically closely related (homologous) strains [19,20,21]. SRH titres >165 mm^2^ were interpreted as consistent with virological protection against heterologous strains [25]. An increase in antibody level of ≥25 mm^2^ was interpreted as a seroconversion [23]. The SRH results for both trials are presented in the Supplementary Data files, in Table S1 (Trial A) and Table S2 (Trial B).

### 3.1. Trial A

**Figure 1:**
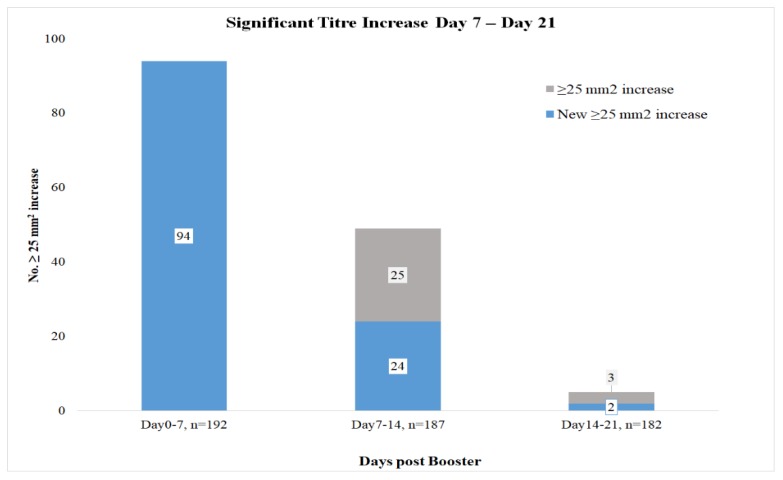
Number of horses with an increase in single radial haemolysis (SRH) titre of ≥25 mm^2^ between the day of booster vaccination (day 0) and day 7 post-vaccination, between day 7 and day 14 post-vaccination, and between day 14 and 21 post-vaccination.

The SRH antibody titre of all horses was measured at the time of booster vaccination and 7, 14, and 21 days post-vaccination. There was a significant increase in SRH titre between the day of the booster vaccination and day 7 post-vaccination (*p* < 0.001), as well as between day 7 and day 14 post-vaccination (*p* < 0.001). There was no significant difference in SRH titre between day 14 and day 21 post vaccination (*p* = 0.43). Ninety-four horses seroconverted between the day of booster vaccination and day 7 post-vaccination, and 49 seroconverted between day 7 and day 14 post-vaccination, but 25 of those had already seroconverted by day 7 (i.e., their titres were continuing to rise). Five horses with SRH titres >143 mm^2^ experienced a significant rise in antibody level between day 14 and day 21 post-vaccination. These results are summarised in Figure 1.

**Figure 2:**
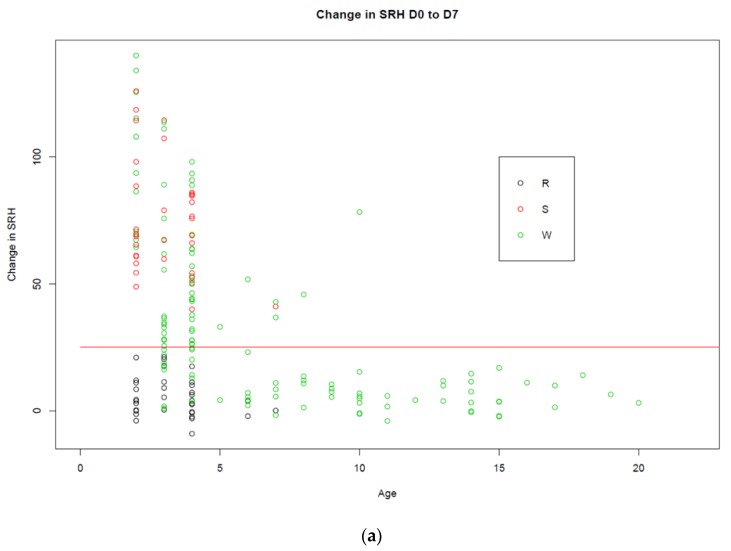

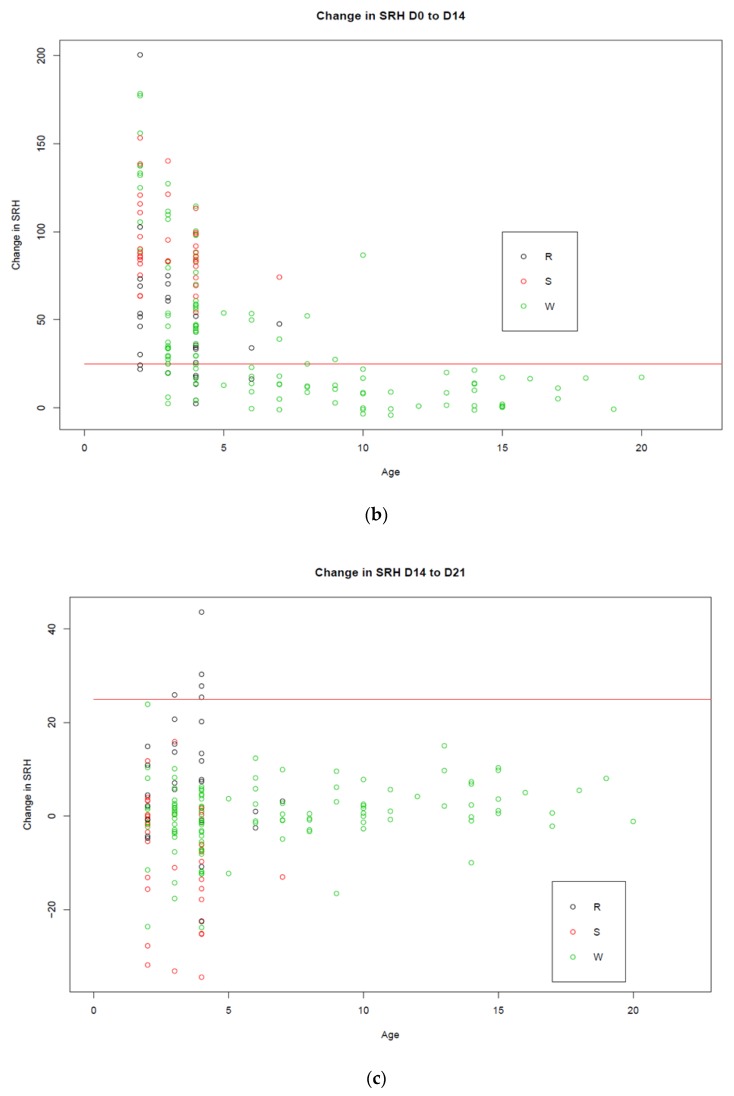
The impact of vaccine type on the response to vaccination. The change in SRH antibody titres between days 0 and 7 (*n* = 192), days 0 and 14 (*n* = 188), and days 14 and 21 (*n* = 182) by age is represented in (**a**–**c**), respectively. The black circles represent the horses vaccinated with a recombinant vaccine (R), the red circles represent the horses vaccinated with a subunit vaccine (S), and the green circles represent horses vaccinated with whole inactivated vaccine (W). The red horizontal line is drawn at 25 mm^2^, as a rise of ≥25 mm^2^ is considered significant (i.e., a seroconversion).

Multivariate analysis demonstrated that age, number of vaccines received and vaccine type all had a significant (*p* < 0.001) impact on the response to booster vaccination. The response to the canary pox recombinant vaccine was delayed when compared to that of the response to the subunit or whole inactivated vaccines. None of the horses vaccinated with the recombinant vaccine seroconverted between day 0 and day 7. The horses that seroconverted during the first week post-booster vaccination were all vaccinated with whole inactivated or subunit vaccines. Twenty two of the 24 horses that initially seroconverted between day 7 and 14 were vaccinated with the recombinant vaccine. The five horses that seroconverted between days 14 and 21 post-vaccination all received the recombinant vaccine. However, three of these had already seroconverted to the vaccine by day 14—i.e., their titres continued to rise between days 14 and 21. The impact of vaccine type on the response to vaccination is illustrated in Figure 2a–c.

**Figure 3:**
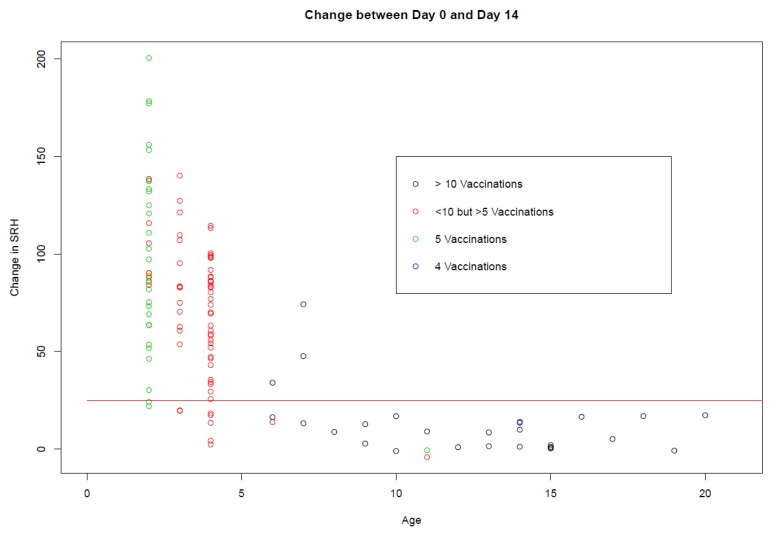
The change in SRH antibody titres between days 0 and 14 (*n* = 123) by age is represented with a red horizontal line drawn at 25 mm^2^, as an increase of ≥ 25 mm^2^ is considered significant (i.e., a seroconversion). The black circles represent the horses that received >10 doses of vaccine, the red circles represent the horses that received <10 but >5 doses of vaccine, the green circles represent horses that received five doses of vaccine, and the blue circles represent horses that received four doses of vaccine. The Argentine horses were not included in the graph, as individual vaccination histories were not available. The number of vaccinations for the Japanese horses illustrated on the graph represent those recorded by the Japanese Racing Association (JRA). Vaccination history prior to JRA ownership was not available; thus, four Japanese horses that did not seroconvert and are represented on the graph as having received <10 doses of vaccine may have actually received more than the number of doses recorded.

As the age of the horse increased, the antibody response to booster vaccination decreased (Figure 2a–c). However, age and number of vaccinations are confounded. This is illustrated in Figure 3, where clearly the majority of horses over four years of age had received more than 10 doses of vaccine and failed to seroconvert to booster vaccination. Only three horses that had received more than 10 doses of vaccine seroconverted in response to booster vaccination. These were one 6-year-old and two 7-year-olds.

**Figure 4:**
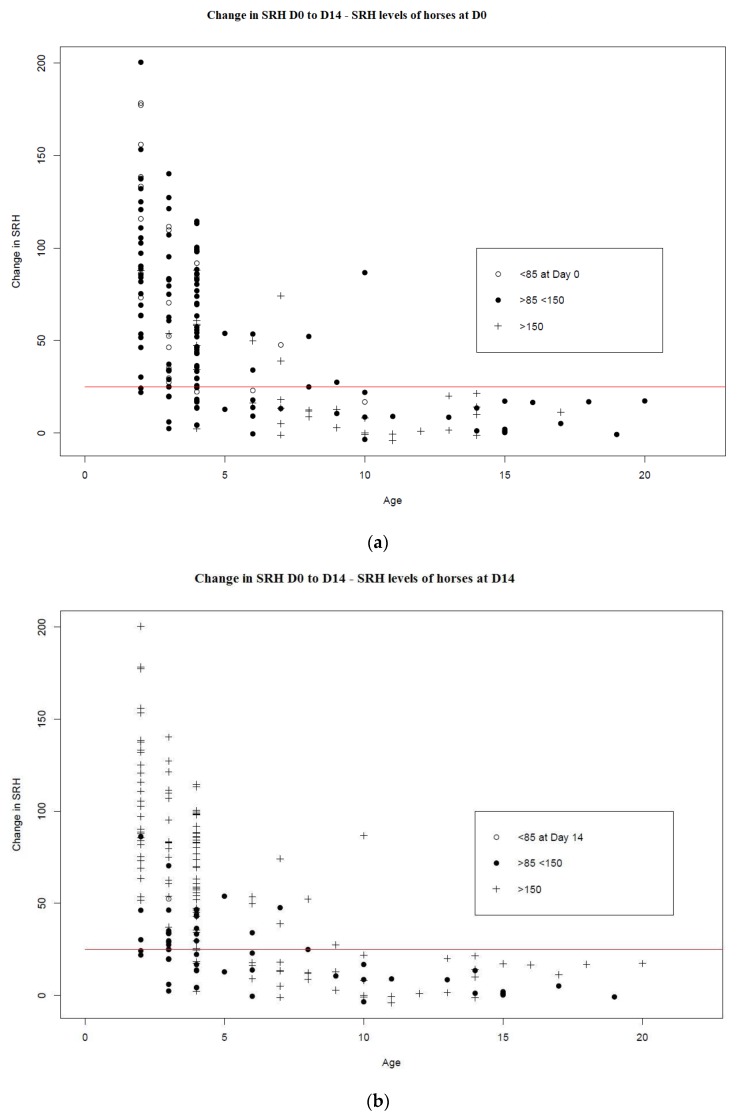
Both Figure 4a,b represent the change in SRH antibody titres between days 0 and 14 (*n* = 188) by age. The red horizontal line is drawn at 25 mm^2^, as an increase of ≥25 mm^2^ is considered significant (i.e., a seroconversion). The figures differ in that (**a**) indicates the titres for the individual horses on day 0, and (**b**) indicates the titres for the same horses on day 14 post-vaccination. The empty circles represent horses with SRH titres <85 mm^2^, the filled circles represent horses with SRH titres >85 mm^2^ but <150 mm^2^,and the crosses represent horses with SRH titres >150 mm^2^.

At time of vaccination, the majority of the horses (74%) with SRH titres >150 mm^2^ were more than four years of age. The majority of horses (75%) with SRH titres >85 mm^2^ and <150 mm^2^ were four years old or younger. Ninety-one percent of the horses with SRH titres <85 mm^2^ were four years old or younger. Fourteen days post-booster vaccination, 67% of horses had SRH titres >150 mm^2^ and only three racehorses (aged three and four years, and one unknown) were below 85 mm^2^. Figure 4 illustrates the SRH titres at time of vaccination (4a) and after vaccination (4b).

### 3.2. Trial B

There was a significant increase in SRH titre between the day of booster vaccination and day 21 (German horses only) or 30 post-vaccination (*p* < 0.001), and significant decrease between days 21/30 and 60, days 60 and 90, and days 90 and 180 post vaccination (*p* < 0.001).

**Figure 5:**
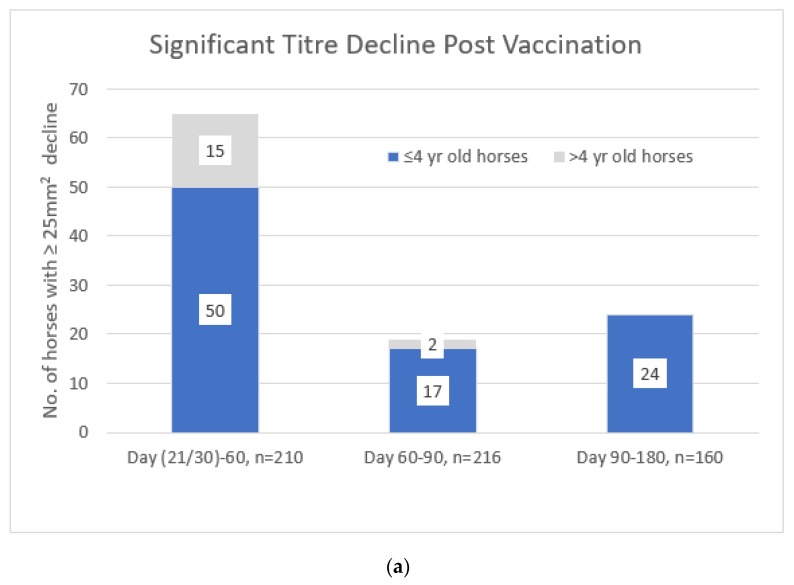

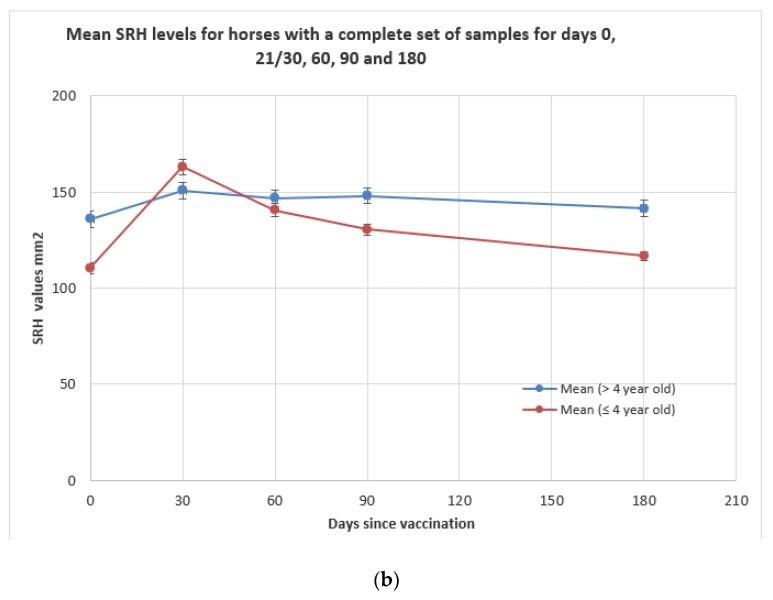
(**a**) The decline in SRH antibody level ≥25 mm^2^ experienced by horses four years old or younger and those over four years old between days 21/30 and 60, days 60 and 90, and days 90 and 180 post-booster vaccination. (**b**) The mean SRH antibody levels for 150 horses (four years old or younger (red), *n* = 96; over four years old (blue), *n* = 54) with a complete set of samples for days 0, 21 (German horses only) or 30 (this sampling is represented on the horizontal axis of the graph as Day 30), 60, 90, and 180.

One hundred and thirty-one of 218 horses (60%) seroconverted between the day of booster vaccination and day 21/30 post vaccination. Between days 21/30 and 60, days 60 and 90, and days 90 and 180 post-vaccination 65, 19, and 24 horses respectively, experienced a decrease in SRH titre of ≥25 mm^2^ (see Figure 5a). This is equal to 31% (210 paired samples), 9% (216 paired samples), and 15% (160 paired samples) of the horses tested at 60, 90, and 180 days post-vaccination, respectively. At least 13 of the 19 and 16 of the 24 horses that experienced a significant decrease in SRH titre between days 60 and 90 and days 90 and 180 post-vaccination respectively, had already experienced a significant decline at a previous time point.

On the day of vaccination, the horses four years old or younger had significantly lower antibody levels than the horses older than four years of age (*p* = 0.003), but they responded very well to vaccination—83% seroconverted by day 21/30. Of the 131 horses that seroconverted post-booster vaccination, 99 were four years of age or younger. At 30 days post-vaccination, the horses four years of age or younger had SRH levels higher than those of the older horses (*p* < 0.001), but it was primarily the young horses that experienced a decline in antibody level by 60, 90, and 180 days post vaccination (see Figure 5a,b).

**Figure 6:**
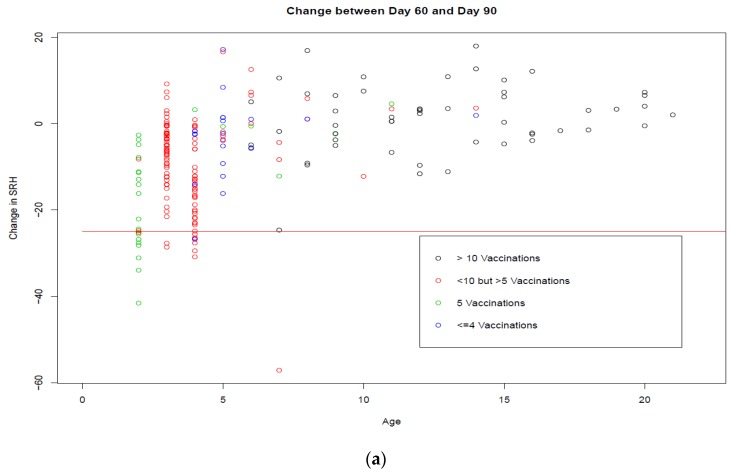

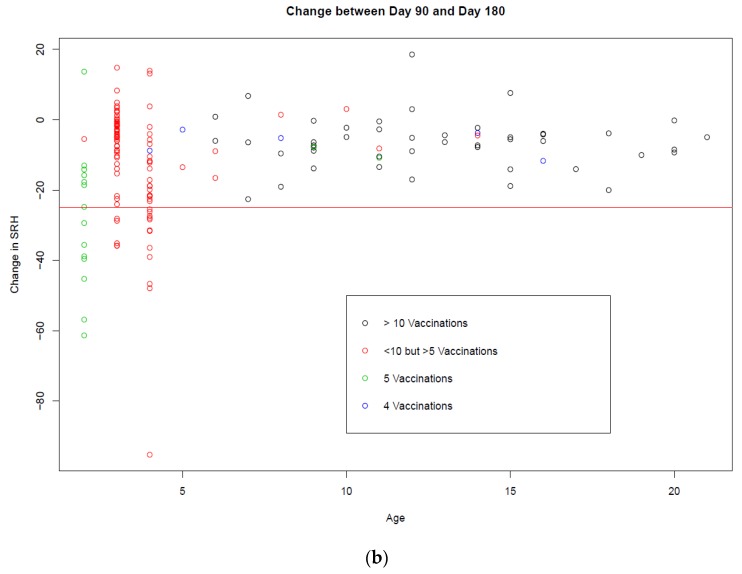
The change in SRH antibody titres between days 60 and 90 (*n* = 216) and days 90 and 180 (*n* = 160) by age is represented in (**a**) and (**b**), respectively. The black circles represent the horses that received >10 doses of vaccine, the red circles represent the horses that received <10 but >5 doses of vaccine, the green circles represent horses that received five doses of vaccine, and the blue circles represent horses that received four doses of vaccine. The red horizontal line is drawn at −25 mm^2^, as a decrease of ≥25 mm^2^ is considered significant.

Multivariate analysis demonstrated that age and the number of vaccines received previously were negatively associated with the initial response to vaccination at days 21/30, but positively associated with the SRH titre at each subsequent time point (*p* < 0.001). However, age and number of vaccines are confounded, as the older horses have received more vaccine doses (Figure 6a,b).

**Figure 7:**
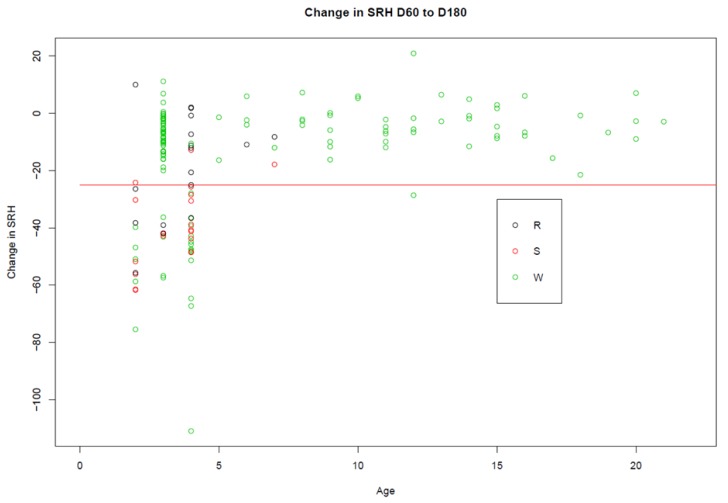
The change in SRH antibody titres between days 60 and 180. The black circles represent the horses vaccinated with the recombinant vaccine (R), the red circles represent the horses vaccinated with the subunit vaccine (S), and the green circles represent horses vaccinated with the whole inactivated vaccine (W). The red horizontal line is drawn at −25 mm^2^, as a decrease of ≥ 25 mm^2^ is considered significant.

The analysis of the effect of time since last vaccination was confounded by the data set; with the exception of the Argentine horses, all horses had been vaccinated approximately six months prior. The analysis of the impact of vaccine type was confounded by age, as only the younger horses received the subunit and recombinant vaccines. However, Figure 7 clearly illustrates that young horses experience a decline in antibody levels between day 60 and day 180 post-booster vaccination irrespective of the type of vaccine used.

SRH antibodies above 165 mm^2^ i.e., levels that are considered consistent with virological protection against heterologous strains [25] are not always achieved by repeated vaccination. By day 180, only 18 of 162 horses had SRH levels above 165 mm^2^. However, despite the decline in antibody levels of the younger horses at day 180, there were only 12 of 162 horses in the study at that time point that had antibody levels <85 mm^2^, which is considered to be the threshold of clinical protection [19,20,21]. Of these, eight were three years old, and the remaining four consisted of a 2-, 4-, 7-, and 16-year-old. When their SRH levels at 60, 90, and 180 days post-vaccination were compared, only two of the 12 had significantly better (i.e., ≥25 mm^2^) levels on day 60 or 90 post-vaccination than they had on day 180. Thus, it appears that for the majority of horses that respond poorly to vaccination, shortening the vaccination interval prior to shipment is of little benefit.

## 4. Discussion

International travel is essential to the sustainability of the horse industry, and horses move between countries for breeding, sales, exhibitions, and competitions. Horses travel more by air than any other species except man, and air transport appears to increase the susceptibility of the respiratory tract to viral infections, such as influenza [26,27,28]. This study was implemented through a public private partnership of the OIE, FEI, and IFHA, with the aim of developing an evidence-based vaccination strategy to decrease the risk of travel-based equine influenza outbreaks. The inclusion of both racehorses and competition horses in the study ensured representation of different sectors of the industry, and the international framework, with participants in three continents, was designed to promote global engagement of stakeholders. Furthermore, vaccines from multinational companies and national companies were included, as were both traditional and second-generation vaccines. With the exception of the Argentine horses, the horses had received their last dose of vaccine approximately six months prior to the study. In accordance with national regulations, the Argentine horses had been vaccinated three months prior to booster vaccination. However, there was no evidence that the time since last vaccination confounded the results, as analysis excluding the Argentine animals from both trials resulted in the same findings.

Vaccine hesitancy, a term that encompasses refusal to vaccinate, delaying vaccination, and lack of confidence, has been identified by the World Health Organisation as one of the top ten global health threats of 2019 [29]. It is important to recognise the spillover of opposition to mandatory vaccination from human to animal health, as horse owners and trainers are concerned about the adverse effects of repeated vaccination [30,31]. Vaccination is one of the most effective means of avoiding equine influenza virus transmission, but in order to retain the confidence of the industry, it is imperative that vaccination regulations are science-based, and desirable that they be harmonised internationally. Currently the OIE, FEI, and IFHA recommendations for vaccination boost before export or competition are not harmonised. The OIE standard is that horses are immunised between 21 and 90 days before shipment. The IFHA recommends that horses are vaccinated during the 60 days immediately prior to export from their country of origin, but not within 14 days of export. For horses competing in FEI events, the last booster must have been given within 6 months and 21 days (and not within 7 days) before arrival at the event. Thus the focus of this investigation was to provide a science-based rationale for which of the intervals used (i.e., 7 days, 14 days, or 21 days) allows horses adequate time to respond to booster vaccination, and whether the immune response elicited is sustainable for 60 days, 90 days, or 6 months (180 days).

The response to booster vaccination in this study was monitored serologically. The induction of a cellular immune response has been demonstrated for the three most widely available types of equine influenza vaccines—whole inactivated, subunit, and canary pox recombinant—all of which were included in the study, but has not yet led to the development of correlates of protection [32,33,34,35]. In contrast, antibodies against the equine influenza haemagglutinin neutralise the virus, and can be measured by SRH or by haemagglutination inhibition. However, only SRH is internationally standardised [36,37], and is currently the method of choice for the assessment of vaccinal immunity dynamics with a view to optimising vaccination regimes. The correlation between SRH antibodies and protection has been observed in experimental challenge studies [38,39,40] and in the field during outbreak investigations [23,25,41]. Antibody levels between 85 mm^2^ and 150 mm^2^, as determined by SRH, are internationally accepted as the protection threshold for clinical protection and antibody levels > 150 mm^2^ for virological protection, when the challenge virus is antigenically closely related to the virus in the vaccine [19,20,21]. However, strain-specific antibodies against influenza are more effective than cross-reactive antibodies [42,43]. Higher antibody levels are required for protection when there is a mismatch between the vaccine strain and the field circulating strain [41,44]. In 1987 in South Africa, where the population had no natural immunity, pre-challenge vaccine-induced SRH levels of >165 mm^2^ correlated with 90% protection against infection, as indicated by seroconversion [25]. The strains in the vaccines were not homologous to the strain that caused the outbreak in South Africa, but all of them induced protective cross-reacting antibodies. Thus, notwithstanding the complexities of strain heterogeneity, the SRH is considered to be the gold standard for the assessment of vaccine efficacy in the absence of virus challenge studies. Although many horses fail to achieve or to maintain virological protection, maximizing their level of neutralizing antibodies decreases virus shedding and reduces the risk of travel-based equine influenza outbreaks.

At the time of commencement of Trial A, the horses had a mean SRH antibody level of 116 mm^2^, and by day 14 post-vaccination the mean had risen to 168 mm^2^. These SRH results are very similar to those observed in a previous study of response to booster vaccination in regularly vaccinated National Hunt racehorses [45]. Trial A focused on comparing the humoral response at 7, 14, and 21 days post-vaccination, and the results suggest that 14 days is the optimum period to allow for horses to respond to vaccination prior to shipment. Seven days is not ideal, as not all horses seroconvert within seven days. In particular, and consistent with a previous comparative vaccine study [45], the response to booster vaccination with the recombinant vaccine was later than the peak antibody response to booster vaccination with the subunit or whole inactivated vaccines. It is important to allow time to respond to the recombinant vaccine, as it is currently one of the vaccines that contains the viruses recommended by the OIE Furthermore, the antibody response to the recombinant vaccine allows for differentiation of infected from vaccinated animals DIVA, [46,47]. The benefit of waiting for 21 days rather than 14 was not significant, as 98% of the horses that seroconverted had done so within 14 days.

Trial B focused on the decrease in antibodies 60, 90, and 180 days post-vaccination, as these were the candidate vaccination intervals for international standardisation. The kinetics of the antibody response differed depending on the age of the horse. This was confounded with the vaccination history, as older horses have received more vaccine doses. Young horses responded better to booster vaccination, but thereafter their antibodies were not as persistent as those of the older horses. The mean SRH antibody level for the 96 horses four years old or younger, with a complete data set, was 141 mm^2^ at day 60, 131 mm^2^ at day 90, and 117 mm^2^ at day 180. The mean SRH antibody level for 54 horses over four years of age was 147 mm^2^ at day 60, 148 mm^2^ at day 90, and 141 mm^2^ at day 180. Thus, the results of the studies in Trial B suggest that for horses that have received four or more doses of vaccine, and are older than four years, there is little benefit in requiring a booster vaccination within 60 or 90 days prior to shipment rather than within 180 days, as their antibody levels are relatively stable. In contrast, the results indicate that there is a potential benefit to younger (four years old or younger) horses in requiring booster vaccination closer to shipment. Eight of the 96 young horses experienced a decline of ≥25 mm^2^ between days 60 and 90, but this rose to 21 between days 90 and 180. Within 90 days prior to shipment is consistent with the current OIE standard. A recommendation of vaccination within 90 days prior to shipment for horses four years old or younger would result in higher antibody levels than a recommendation for vaccination within 180 days prior to shipment. This study did not include full data sets from horses that had received fewer than four doses of vaccine, and as previous studies provided evidence of poor antibody persistence after initial vaccinations, it would be prudent to include such horses, irrespective of their age, in the recommendation for vaccination within 90 days prior to shipment [48].

International horse movement is under the control of national veterinary authorities, not equestrian authorities. The majority of countries that insist on vaccination against equine influenza require that the horse receives a vaccine between 14 and 90 days before shipment. Equipped with the information derived from this study, importing countries can gain insight into different vaccination strategies, taking account of the time required to respond to different types of vaccines and the persistence of vaccine-induced antibodies with increasing age. This in turn will hopefully contribute to a harmonised approach in line with revised OIE guidelines.

## Figures and Tables

**Table 1:** Recommendations for equine influenza vaccination.

Recommendation	FEI ^1^	IFHA ^2^	OIE ^3^
Boost before export/competition	For horses competing, the last booster must be given within 6 months + 21 days (and not within 7 days) before arrival at the event	During the 60 days immediately prior to export from its country of origin, but not within 14 days of export.	Immunised between 21 and 90 days before shipment

^1^ Federation Equestre Internationale. ^2^ International Federation for Horseracing Authorities. ^3^ World Organisation for Animal Health (Office International des Epizooties).

## Supplementary Materials

The following are available online at https://www.mdpi.com/2076-393X/8/1/107/s1, Table S1: Supplementary data for Trial A, Table S2: Supplementary data for Trial B.
